# Response to febuxostat according to clinical subtypes of hyperuricemia: a prospective cohort study in primary gout

**DOI:** 10.1186/s13075-023-03228-y

**Published:** 2023-12-11

**Authors:** Han Qi, Mingshu Sun, Robert Terkeltaub, Xiaomei Xue, Xinde Li, Lingling Cui, Yuwei He, Fei Yan, Ruixia Sun, Ying Chen, Zhaotong Jia, Xiaoyu Cheng, Lidan Ma, Tian Liu, Nicola Dalbeth, Changgui Li

**Affiliations:** 1 Department of Endocrinology and Metabolism, the Affiliated Hospital of Qingdao University, Qingdao, China; 2 Shandong Provincial Key Laboratory of Metabolic Diseases and Qingdao Key Laboratory of Gout, the Affiliated Hospital of Qingdao University, Qingdao, China; 3 Institute of Metabolic Diseases, Qingdao University, Qingdao, China; 4 Shandong Provincial Clinical Research Center for Immune Diseases and Gout, Qingdao, China; 5 Department of Rheumatology, the Affiliated Hospital of Qingdao University, Qingdao, China; 6 VA San Diego VA Healthcare Center, University of California San Diego, La Jolla, CA USA; 7 Department of Medicine, University of Auckland, Auckland, New Zealand

**Keywords:** Gout, Febuxostat, Clinical subtypes of hyperuricemia

## Abstract

**Background:**

While xanthine oxidase inhibitors target uric acid production, renal urate underexcretion is the predominant subtypes in gout. This study was to compare treatment response to the XOI febuxostat in a gout cohort according to clinical subtypes of hyperuricemia.

**Methods:**

A prospective cohort study was conducted to compare the efficacy and safety of febuxostat (initially 20 mg daily, escalating to 40 mg daily if not at target) in 644 gout patients with the three major clinical subtypes for 12 weeks. Hyperuricemia was defined as the renal overload subtype, the renal underexcretion subtype, or the combined subtype based on UUE > or ≤ 600 mg/d/1.73 m^2^ and FE_UA_ < or ≥ 5.5%. The primary endpoint was the rate of achieving serum urate (SU) < 6 mg/dL at week 12.

**Results:**

Fewer participants with combined subtype achieved the SU target, 45.5% compared with 64.8% with overload subtype (*P* = 0.007), and 56.6% with underexcretion subtype (*P* = 0.022). More participants with combined subtype (82%) had febuxostat escalated to 40 mg than those with overload (62%, *P* = 0.001) or underexcretion subtype (68%, *P* = 0.001). In all participants, combined subtype hyperuricemia (OR = 0.64, 95%CI 0.41–0.99, *P* = 0.048) and baseline SU (OR = 0.74, 95%CI 0.62–0.89, *P* = 0.001) were independently associated with lower rates of achieving SU target.

**Conclusions:**

People with combined subtype have a lower response to febuxostat, compared to those with either overload or underexcretion subtype. Assessment of hyperuricemia subtype may provide useful clinical data in predicting febuxostat response.

**Supplementary Information:**

The online version contains supplementary material available at 10.1186/s13075-023-03228-y.

## Key Message

• Combined subtype of hyperuricemia had lower urate-lowering response to febuxostat compared with other subtypes.

• Typing as combined subtype and baseline serum urate were independent risk factors for targeting.

• Assessment of hyperuricemia clinical subtype may provide useful data in predicting febuxostat response.

**Supplementary Information:**

The online version contains supplementary material available at 10.1186/s13075-023-03228-y.

## Background

Gout is a common inflammatory condition caused by monosodium urate crystal deposition due to elevated serum urate (SU) concentration (hyperuricemia) [1]. The SU concentration is determined by a balance between urate production and elimination. Hyperuricemia has been defined as three clinical subtypes: renal urate overload, renal urate underexcretion, or combined type, based on both urinary urate excretion (UUE) and uric acid fractional excretion (FE_UA_) [2–4]. Primary therapy of gout is the treat-to-target approach of urate-lowering drug with dose titration to achieve a SU target, which is < 6 mg/dL for most patients [5–7].

Gout management guidelines from the American College of Rheumatology (ACR) and European Alliance of Associations for Rheumatology (EULAR) recommend allopurinol, the xanthine oxidase inhibitor (XOI), as the first line urate-lowering treatment (ULT) medicine [5, 6]. Given the high prevalence of HLA-B*5801 in Asian people, the Asia–Pacific League of Associations for Rheumatology (APLAR) guideline recommends febuxostat, another XOI which is broadly used in Asia, equally to avoid allopurinol-induced hypersensitivity syndrome [8, 9]. Uricosuric medications such as benzbromarone and probenecid represent a further class of ULT; these agents inhibit uric acid reabsorption in the proximal renal tubule through effects on URAT1 and other urate transporters [10–12]. Uricosuric drugs are recommended as second line ULT medicine in most countries. Theoretically, drugs selected based on their pathogenic mechanism of action might be more rational in terms of efficacy and the potential for lower effective doses. However, current gout management guidelines do not recommend ULT drug selection based on the type of hyperuricemia due to insufficient evidence [5, 6].

Accumulating clinical data suggest that selecting ULT agent according to clinical subtypes may allow a more personalized approach to gout management. In a prospective study of 86 participants with primary gout, benzbromarone showed greater SU reduction in those with uric acid underexcretion compared to those with normal excretion, and the proportion of participants achieving SU < 6.0 mg/dL was higher with ULT drug selection according to renal urate excretion [13, 14]. Our prior prospective cohort study of 220 gout patients also showed that fixed low-dose benzbromarone was more effective in participants with renal urate underexcretion than in untyped gout patients [13, 14]. Meanwhile, post hoc analysis of a multicenter, randomized, double-blind, placebo-controlled trial of 153 gout patients revealed a better response to the XOI febuxostat in participants with overproduction type compared with underexcretion type [15]. We further conducted a comparative effectiveness clinical trial of low-dose benzbromarone versus low-dose febuxostat in gout patients with renal urate underexcretion, in which benzbromarone had superior urate-lowering efficacy and similar safety [16]. Taken together, these studies suggest potential benefit for taking a pathogenic approach when selecting ULT agent. However, it remains unclear whether a typing-based ULT strategy can be applied to all people with gout.

In addition to urate-regulating molecular pathways, other mechanisms might also contribute to the clinical traits. About 50% of gout patients have metabolic syndrome (MetS) or its components like obesity, fatty liver disease, hypertension, and diabetes [17, 18]. MetS and insulin resistance have been proved to induce renal urate clearance and uric acid de novo synthesis [19–22]. Whether or not the mechanism is involved in this type has not been documented. Furthermore, no available literature compared the ULT responses between the three types.

To investigate whether the clinical subtypes can influence treatment response to the XOI febuxostat, we performed a 12-week, prospective cohort study to compare efficacy and safety of febuxostat dose escalation (20 mg to 40 mg daily) in gout patients assessed as renal urate overload, renal urate underexcretion and combined type. We hypothesized that responses to febuxostat would differ according to the type of hyperuricemia.

## Methods

### Study design and participants

A prospective cohort study was conducted to compare the efficacy and safety of febuxostat dose escalation to achieve the target SU < 6 mg/dL in people with primary gout for 12 weeks. Participants were recruited between February 2021 and December 2021 in the Gout Clinic of the Affiliated Hospital of QingDao University. All participants had gout diagnosed according to the 2015 ACR/EULAR gout classification criteria [23] and were with identified clinical subtypes of hyperuricemia (data blinded to the prescribing administrators). Hyperuricemia subtypes were defined as the renal overload subtype when UUE > 600 mg/d/1.73m^2^ and FE_UA_ ≥ 5.5%, the renal underexcretion subtype when UUE ≤ 600 mg/d/1.73m^2^ and FE_UA_ < 5.5%, or the combined subtype when UUE > 600 mg/d/1.73m^2^ and FE_UA_ < 5.5% [2–4], assessed from 24-h urine samples. Participants were followed at baseline and every 4 weeks until 12 weeks. At week 16, all participants were followed for safety evaluation.

Inclusion criteria were men with primary gout and SU ≥ 7.0 mg/dL, without any urate-lowing drugs in the preceding 1 month before enrollment [7]. All participants had hyperuricemia typing before entry into the study. Exclusion criteria were allergy to febuxostat, gout flare in the preceding 2 weeks, alanine aminotransferase or aspartate aminotransferase 1.5 times higher than the upper normal limit, eGFR < 60 ml/min/1.73 m^2^, or patients with heart failure due to any case with worse than Class I New York Heart Association classification to avoid confounding factors to the primary outcome. According to previous reports, eGFR was inversely associated with fractional excretion of urinary uric acid (FE_UA_) [24–26]. So, we assumed that CKD ≥ stage 3 might be a confounder for hyperuricemia classification, serving as a secondary etiological factor of hyperuricemia, in addition to primary pathophysiological mechanisms. Those patients were excluded to avoid inaccurate definition of hyperuricemia type.

The study was approved by the Ethics Committee of the Affiliated Hospital of QingDao University and was registered at the China Clinical Trial Registration Center (ChiCTR2100043573). Written informed consent was obtained from all participants.

### Treatment and follow-up procedures

Before the start of the trial, all participants were required to undergo a 14-day washout period to withdraw any drugs affecting SU levels such as dihydrochlorothiazide and follow a low-purine diet (Supplementary Table 1) before baseline data collected. Febuxostat was administered to all participants at the initiation dose of 20 mg every morning and escalated to 40 mg if SU > 6 mg/dL during the follow-up visits. No anti-inflammatory prophylaxis was used. For participants experienced gout flare [27], etoricoxib or colchicine was given on demand. For participants with transaminases elevated up to 1.5 times of baseline level, hepatoprotective medicine (diammonium glycyrrhizinate, silibinin, or polyene phosphatidylcholine) was prescribed. Other urate-lowering drugs or drugs with potent urate-lowering activity were not allowed during the study.

We took several measures to ensure compliance. Study procedures, detailed dietary instructions, and other information were provided to patients through a WeChat channel. A patient diary was provided for patients to record their daily medications. This diary was reviewed at each study visit.

Baseline information were acquired at face-to-face study visits including age, disease duration, body mass index (BMI, kg/m^2^), systolic blood pressure (SBP), diastolic blood pressure (DBP), the presence or absence of subcutaneous tophi, and associated disorders such as hypertension, diabetes, fatty liver disease, and hyperlipidemia. Fasting blood was collected and biochemical parameters include serum concentrations of urate (SU), blood glucose (Glu), triglyceride (TG), total cholesterol (TC), high-density lipoprotein-cholesterol (HDL-C), creatinine (Cr), alanine aminotransferase (ALT), and aspartate aminotransferase (AST) were tested using an automatic biochemical analyzer (TBA-40FR; TOSHIBA, Japan).

The FE_UA_ and 24-h UUE were calculated by 24-h urine volume and 24-h uUA and uCr. FE_UA_ = uUA/uCr × sCr/sUA × 100% and UUE = uUA × 24-h urinary volume / [0.0061 × height (cm) + 0.0128 × weight (kg) – 0.1529) × 1.73 (mg/d/1.73m2)] [28]. Kidney function was assessed using estimated glomerular filtration rate (eGFR) (CKD-EPI design formulas) [29]. The metabolic syndrome was defined according to the Chinese Diabetes Society criteria as the presence of three or more of the four components: (1) BMI ≥ 25 kg/m^2^; (2) fasting Glu ≥ 6.1 mmol/L or 2 h postprandial Glu ≥ 7.8 mmol/L; (3) blood pressure ≥ 140/90 mm Hg; or (4) fasting serum TG ≥ 1.7 mmol/L, or HDL-C < 0.9 mmol/L in men or < 1.0 mmol/L in women. Individuals who had been diagnosed with hypertension or diabetes and used antihypertensive or antidiabetic medications met the criteria for hypertension or diabetes [30]. Insulin resistance was calculated with the Triglyceride/HDL-C ratio [31]. Insulin was evaluated using an automated enzyme immunoassay system analyzer.

### Outcomes

The primary efficacy outcome was the proportion of participants achieving the target SU < 6 mg/dL at week 12. The proportion of participants achieving SU < 5 mg/dL at week 12, proportion of participants requiring febuxostat 40 mg daily, predictors of febuxostat response for all participants, and for each hyperuricemia subtype were also investigated. Safety outcomes were gout flare incidence defined as the percentage of participants who experienced patient-reported flare with pain visual analog score > 3 of a 0–10 scale [27], changes in renal function, and the percentage of participants who needed hepatoprotective medicine and newly onset cerebrovascular disease diagnosed by specialist physicians throughout the study period and the following 4 weeks.

### Sample size

The sample size for the prospective study was determined based on the primary endpoint (the rate of achieving target SU < 6.0 mg/dL after 12 weeks). We assumed that SU target with febuxostat 40 mg daily would be achieved in about 70% of participants with renal overload type and 50% of participants with renal underexcretion type based on a previous study [15]. The sampling ratio of the three hyperuricemia types was set at 10% renal overload type: 60% renal underexcretion type: 25% combined type based on a gout cohort of 3578 participants (unpublished data from other patients in our clinic). To achieve a 5% two-sided significance level and 80% power to detect the differences between the three types, we calculated 52 participants were required for the renal overload type, 312 participants for the renal underexcretion type, and 130 participants for the combined type. A sample size of 65, 390, and 163 for each group was calculated account for an estimated 20% dropout rate.

### Statistical analysis

All statistical analyses were analyzed by SPSS 25.0 and GraphPad prism 9.0 software. Continuous variables were presented as mean (standard deviation, SD) or median (interquartile range, IQR), and categorical variables as frequency. Pearson* χ*^2^ test was applied in categorical variables for group comparison. Independent samples *t*-test, one-way analysis of variance (ANOVA), or Mann–Whitney *U* was used to compare continuous variables. In the end point analysis, the statistical analysis was corrected for multiple comparisons. This study used both in-tentation-to-treat (ITT) analysis and per-protocol (PP) analysis for the primary outcome. Binary logistic regression was used to assess predictors of SU target achievement in univariable and multivariable analyses. Body mass index, age, disease duration, baseline SU, and other variables with *P* < 0.2 in univariable analysis were included in multiple logistic regression analyses. All tests were two-sided. Statistical significance was at *P* < 0.05.

## Results

### Participants and baseline characteristics

**Fig. 1:**
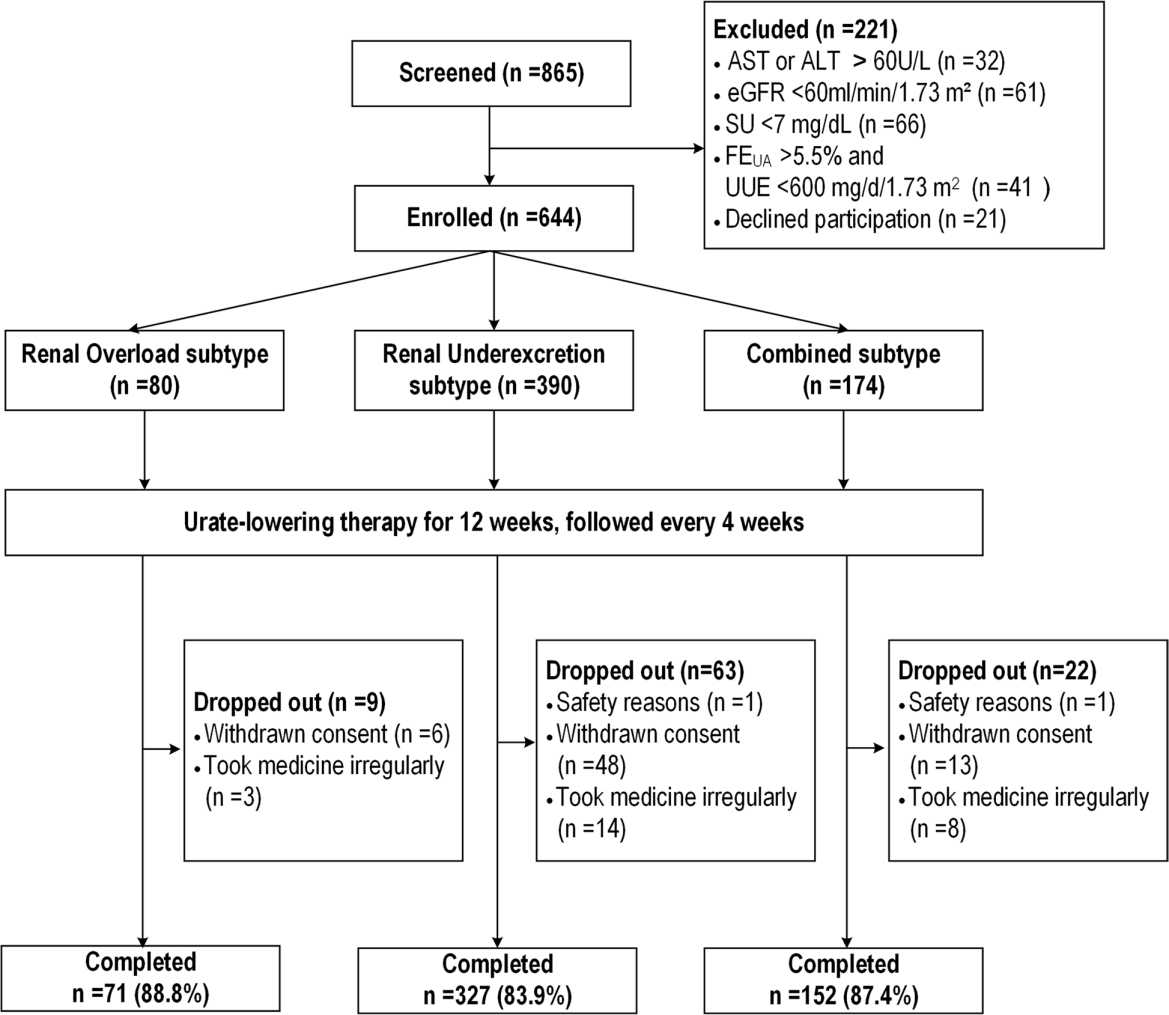
Participant flow throughout study

A total of 865 participants were screened; 644 participants were enrolled. A small portion of patients were excluded from this study because of eGFR < 60 ml/min/1.73m^2^ (*n* = 61, 7.1%); 550 participants completed the study and were included in the outcome analyses (Fig. 1). Of the 94 participants who dropped out, the reasons were 67 withdrew their consent, 25 protocol violations with missing doses, and 2 participants were because of safety reasons (see “Safety analysis”).

Of the 644 participants enrolled, 80 (12.4%) had renal overload type, 390 (60.6%) had renal underexcretion type, and 174 (27.0%) had combined type. Clinical characteristics of all enrolled participants at baseline are shown in Table 1. Participants with renal overload type were older and with lower SU level compared with participants with the other two subtypes (*P* < 0.05). More participants with combined type were with obesity, hyperlipidemia, NAFLD, and the TG/HDL-C ratio, compared with participants with the other two subtypes (*P* < 0.05). More detailed baseline features are presented in Table 1.

**Table 1:** Demographic and baseline clinical characteristics of participants

Parameters	All Participants (*n* = 644)	Hyperuricemia subtype			
		Renal overload (*n* = 80)	Renal underexcretion (*n* = 390)	Combined (*n* = 174)	*P*-value
Demographic and general characteristics					
Age, years	45.39 (12.21)	50.05 (14.62)	44.50 (15.23) *	42.59 (14.23) *	< *0.001*
BMI, kg/m^2^	27.53 (3.63)	26.56 (3.05)	27.26 (3.66)	28.50 (3.62) **^##^	< *0.001*
SBP, mmHg	135 (124,147)	137 (126,148)	135 (124,147)	136 (125,149)	0.458
DBP, mmHg	86 (79,94)	88 (81,96)	87 (81,95)	86 (75,94)	0.327
Biochemical parameters					
Blood glucose, mmol/L	5.75 (0.71)	6.02 (0.86)	5.69 (0.75) **	5.77 (0.74) *	*0.002*
Total cholesterol, mmol/L	4.98 (0.93)	4.99 (0.96)	4.98 (0.95)	4.97 (0.84)	0.981
Triglyceride, mmol/L	2.14 (1.44)	1.78 (0.99)	2.11 (1.49)	2.32 (1.51) *	*0.022*
HDL-C, mmol/L	1.08 (0.31)	1.13 (0.36)	1.09 (0.33)	1.04 (0.27) *	0.077
ALT, U/L	25 (18,37)	23 (19.30)	24 (17.34)	29 (21.43) **^##^	< *0.001*
AST, U/L	20 (17,25)	21 (16.24)	20 (17.25)	21 (17.26)	0.414
Creatinine, μmol/L	86.36 (12.64)	86.46 (13.26)	87.58 (12.64)	83.80 (12.65) ^#^	*0.012*
eGFR, ml/min/1.73 m^2^	92.83 (18.98)	89.92 (19.02)	91.54 (19.10)	96.81 (17.79) *^#^	*0.002*
Urine pH	5.61 (0.45)	5.77 (0.49)	5.58 (0.45)	5.64 (0.44)	0.675
Gout characteristics					
Disease duration, years	7.83 (5.83)	8.65 (7.35)	7.94 (6.01)	7.22 (4.44)	0.407
Family history of gout, *n* (%)	149 (23.1)	22 (27.5)	85 (21.8)	42 (24.1)	0.873
Tophi, *n* (%)	99 (15.4)	15(18.8)	57 (14.6)	27 (15.5)	0.765
Serum urate, mg/dL	9.42 (1.39)	8.60 (1.33)	9.47 (1.37) **	9.70 (1.30) **	< *0.001*
FE_UA,_ %	4.30 (0.63)	6.59 (1.24)	3.74 (0.85)	4.27 (1.27)	< *0.001*
UUE, mg/d/1.73 m^2^	600.00 (226.18)	846.59 (268.55)	452.58 (112.79)	741.18 (157.70)	< *0.001*
Coexisting conditions					
Diabetes, *n* (%)	64 (10.00)	13 (16.3)	29 (7.4) *	22 (12.6) ^#^	*0.001*
Hypertension, *n* (%)	232 (36.0)	32 (40.0)	135 (34.6)	65 (38.5)	0.401
TG/HDL-C	1.60 (1.04, 2.48)	1.30 (0.91, 2.06)	1.58 (1.04, 2.42) *	1.89 (1.15, 2,76) *^#^	*0.001*
Fasting insulin, μU/mL	12.43 (8.50, 17.80)	11.75 (8.06, 18.48)	11.33 (8.19, 16.74)	14.48 (10.28, 22.47) *^#^	*0.002*
NAFLD, *n* (%)	124 (20.0)	10 (12.50)	61 (15.64)	51 (29.31) **^##^	< *0.001*
Hyperlipidemia, *n* (%)	183 (28.4)	19 (23.8)	98 (25.1)	66 (37.9) *^#^	*0.038*
Obesity, *n* (%)	263 (42.4)	26 (33.8)	146 (39.5)	91 (52.6) *^#^	*0.003*
Metabolic syndrome, *n* (%)	221 (34.3)	27 (33.8)	118 (30.3)	76 (43.7) ^#^	*0.008*

Data are showed as mean (standard deviation) or median (interquartile range) as appropriate

*BMI* body mass index, *SBP* systolic blood pressure, *DBP* diastolic blood pressure, *TG* triglyceride, *HDL-C* high-density lipoprotein-cholesterol, *ALT* alanine aminotransferase, *AST* aspartate aminotransferase, *eGFR* estimated glomerular filtration rate, *ZJU index* Zhejiang University index, *HOMA-IR* homeostatic model assessment of insulin resistance, *FE*_*UA*_ fractional excretion of urinary uric acid, *UUE* 24-h urinary urate excretion, *NAFLD* non-alcoholic fatty liver disease

^*^Compared with renal overload group, *P* < 0.05

^**^Compared with renal overload group, *P* < 0.001

^#^Compared with renal underexcretion group, *P* < 0.05

^##^Compared with renal underexcretion group,* P* < 0.001

### Serum urate lowering efficacy

**Fig. 2:**
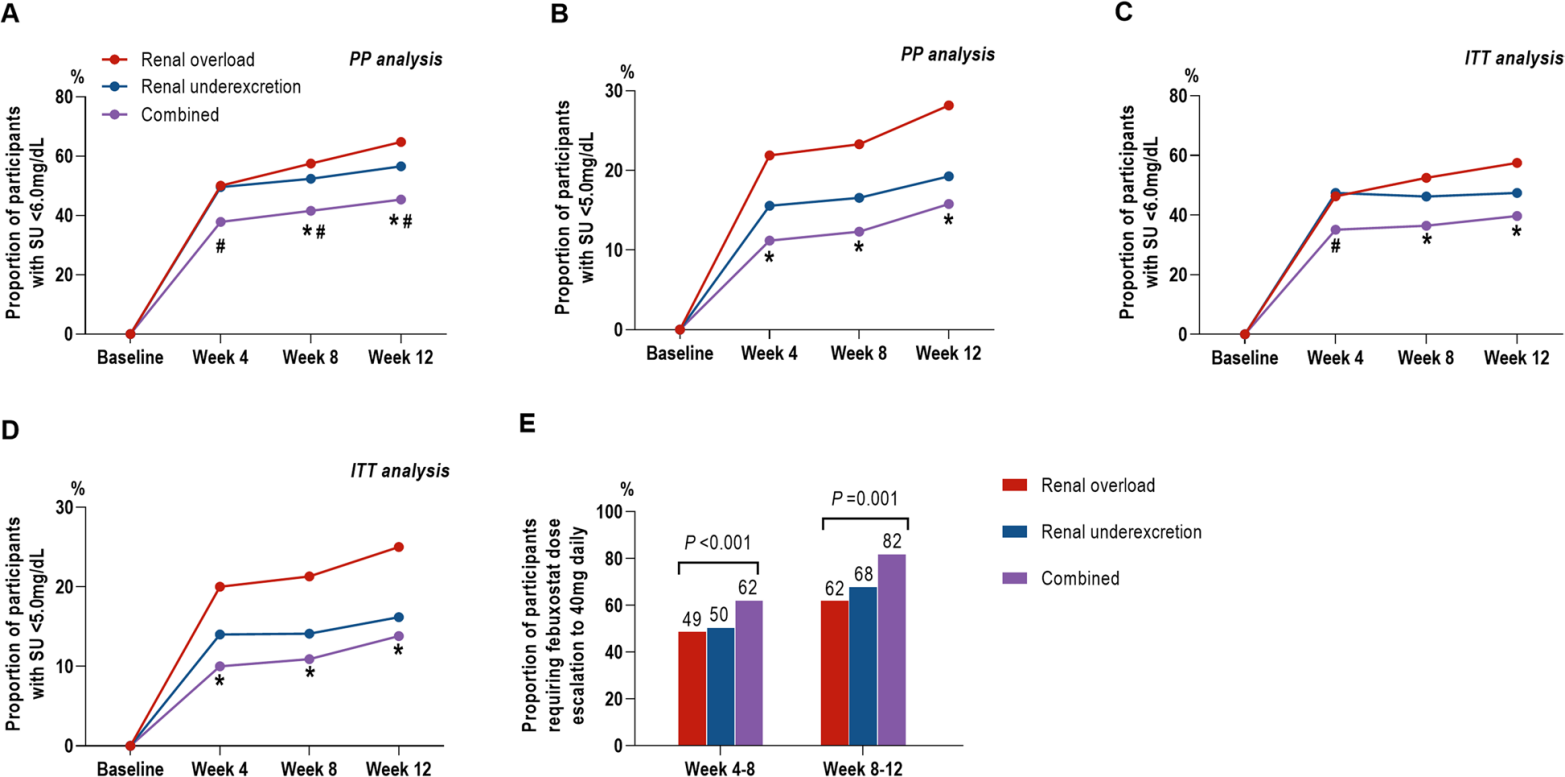
Serum urate lowering efficacy of febuxostat according to subtype of hyperuricemia. **A** Proportion of participants with SU < 6.0 mg/dL at weeks 4, 8, and 12 using per-protocol analysis. **B** Proportion of SU < 5.0 mg/dL at weeks 4, 8, and 12 using per-protocol analysis.** C** Proportion of participants with SU < 6.0 mg/dL at weeks 4, 8, and 12 using intention-to-treat analysis. **D** Proportion of SU < 5.0 mg/dL at weeks 4, 8, and 12 using intention-to-treat analysis. **E** Proportion of participants requiring febuxostat dose escalation to 40 mg daily. PP per-protocol, ITT intention-to-treat. Asterisk indicates compared with the renal overload subtype, *P* < 0.05, number sign indicates compared with the renal underexcretion subtype, *P* < 0.05

A total of 550 participants completed the study and 300 (54.5%) participants achieved SU < 6.0 mg/dL after 12 weeks ULT. According to the PP analysis, the proportion of participants achieving target differed in the three groups: 64.8% with renal overload subtype, 56.6% with underexcretion subtype, and 45.5% with combined subtype (*P* = 0.013). The proportion of participants achieving target was similar between renal overload subtype (50.0% at week 4, 57.5% at week 8, and 64.8% at week 12) and underexcretion subtype (49.6% at week 4, 52.4% at week 8, and 56.6% at week 12) (*P* > 0.05 at each timepoint). More participants with overload subtype achieved the SU target than participants with combined subtype at week 8 (57.5% vs*.* 41.6%, *P* = 0.024) and week 12 (64.8% vs. 45.4%, *P* = 0.007), and more participants with renal underexcretion subtype achieved the SU target than participants with combined subtype at week 4 (49.6% vs*.* 37.8%, *P* = 0.012), week 8 (52.4% vs*.* 41.6%, *P* = 0.026), and week 12 (56.6% vs*.* 45.4%, *P* = 0.022) (Fig. 2A).

The proportion of participants achieving a lower SU level of < 5.0 mg/dL was higher in participants with renal overload subtype than participants with combined subtype at week 4 (21.9% vs. 11.2%,* P* = 0.031), week 8 (23.3% vs*.* 12.2%, *P* = 0.035), and week 12 (28.2% vs*.* 15.8%, *P* = 0.030). For the lower SU level, there was no significant difference between participants with renal overload and underexcretion subtypes or between participants with combined and underexcretion subtype at any timepoint (Fig. 2B).

In the ITT analysis, more participants with overload subtype achieved the SU target than participants with combined subtype at week 8 (52.5% vs*.* 36.4%, *P* = 0.027) and week 12 (57.5% vs*.* 39.7%, *P* = 0.026) (Fig. 2C), while no difference was observed between underexcretion subtype and combined subtype at week 8 (46.2% vs*.* 36.4%, *P* > 0.05) and at week 12 (47.4% vs*.* 39.7%, *P* > 0.05). The proportion of participants achieving a lower SU level of < 5.0 mg/dL was higher in participants with renal overload subtype than participants with combined subtype at week 4 (20.0% vs*.* 10.0%,* P* = 0.039), week 8 (21.3% vs*.* 10.9%, *P* = 0.028), and week 12 (25.0% vs*.* 13.8%, *P* = 0.030) (Fig. 2D). The proportion of participants requiring febuxostat escalation to 40 mg was higher in participants with combined type than in participants with renal overload type (62.2% vs*.* 49.0% at week 8, *P* = 0.066, 82% vs*.* 62% at week 12, *P* = 0.001) or with underexcretion type (62.2% vs*.* 50.4%, *P* = 0.012 at week 8; 82% vs*.* 68% at week 12, *P* = 0.001) (Fig. 2E).

### Predictors of serum urate lowering response

**Fig. 3:**
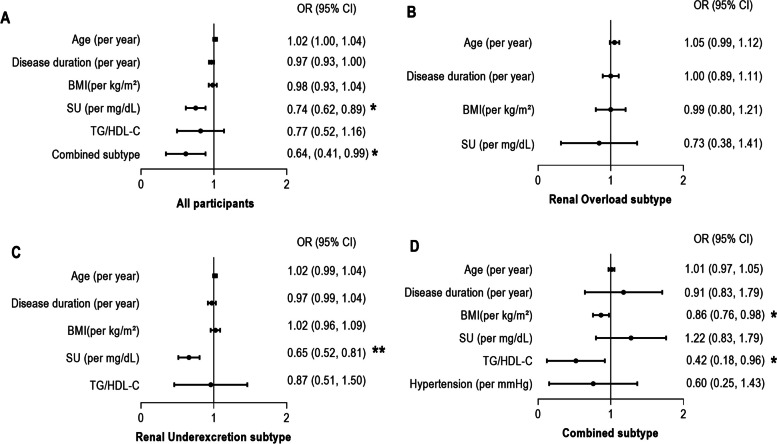
Odds ratio (OR) (95% CI) of baseline predictors associated with achieving SU target (SU < 6.0 mg/dL) in multivariable analysis. **A** All participants. **B** Participants with renal overload subtype. **C** Participants with renal underexcretion subtype. **D** Participants with combined subtype

In multiple logistic regression analyses of all participants in the longitudinal study (Table 2), typing as combined type (OR = 0.64, 95%CI 0.41–0.99, *P* = 0.048) and baseline SU (OR = 0.74, 95%CI 0.62–0.89, *P* = 0.001) were independently associated with lower rates of achieving SU target (Fig. 3A). For participants with renal overload type, no predictor was associated with achieving SU target (Fig. 3B). For participants with renal underexcretion type, high baseline SU independently predicted lower rates of achieving SU target (OR = 0.65, 95%CI 0.52–0.81, *P* < 0.001) (Fig. 3C). For participants with combined type, high baseline BMI (OR = 0.86, 95%CI 0.76–0.98, *P* = 0.018) and high TG/HDL-C ratio (OR = 0.42, 95%CI 0.18–0.96, *P* = 0.039) were independently associated with lower rates of achieving SU target (Fig. 3D).

**Table 2:** Baseline clinical variables associated with the SU target achievement

Indicators	Univariable logistic regression model			Multivariate logistic regression model		
	**OR**	**95% CI**	***P-*value**	**OR**	**95% CI**	***P-*value**
All participants						
Age, per year	1.02	(1.01, 1.04)	< 0.001	1.02	(1.00, 1.04)	0.086
Disease duration, per year	0.99	(0.96, 1.02)	0.584	0.97	(0.93, 1.00)	0.123
BMI, per kg/m^2^	0.95	(0.91, 1.00)	0.044	0.98	(0.93, 1.04)	0.524
Serum urate, per mg/dL	0.66	(0.57, 0.76)	< 0.001	0.74	(0.62, 0.89)	*0.001*
Blood glucose, per mmol/L	1.00	(0.81, 1.24)	1.000			
eGFR, per ml/min/1.73 m^2^	0.97	(0.99, 1.00)	0.972			
TG/HDL-C, per unit	0.65	(0.47, 0.91)	0.014	0.77	(0.52, 1.16)	0.217
Hypertension	1.00	(0.71, 1.4)	0.987			
Fatty liver disease	1.06	(0.68, 1.64)	0.806			
Combined type hyperuricemia	0.60	(0.41, 0.88)	0.008	0.64	(0.41, 0.99)	*0.048*
Renal overload subtype						
Age, per year	1.05	(0.99, 1.10)	0.070	1.05	(0.99, 1.12)	0.107
Disease duration, per year	1.02	(0.93, 1.11)	0.665	1.00	(0.89, 1.11)	0.937
BMI, per kg/m^2^	0.95	(0.81, 1.13)	0.560	0.99	(0.80, 1.21)	0.908
Serum urate, per mg/dL	0.65	(0.39, 1.08)	0.096	0.73	(0.38, 1.41)	0.731
Blood glucose, per mmol/L	0.74	(0.43, 1.27)	0.272			
eGFR, per ml/min/1.73 m^2^	1.00	(0.98, 1.04)	0.717			
TG/HDL-C, per unit	0.93	(0.33, 2.65)	0.892			
Hypertension	0.75	(0.28, 2.01)	0.562			
Fatty liver disease	0.64	(0.16, 2.64)	0.537			
Renal underexcretion subtype						
Age, per year	1.02	(1.01, 1.04)	0.008	1.02	(0.99, 1.04)	0.248
Disease duration, per year	0.99	(0.96, 1.03)	0.666	0.97	(0.92, 1.03)	0.306
BMI, per kg/m^2^	0.99	(0.94, 1.05)	0.761	1.02	(0.96, 1.09)	0.515
Serum urate, per mg/dL	0.61	(0.51, 0.74)	< 0.001	0.65	(0.52, 0.81)	< *0.001*
Blood glucose, per mmol/L	1.08	(0.82, 1.44)	0.583			
eGFR, per ml/min/1.73 m^2^	1.00	(0.99, 1.01)	0.714			
TG/HDL-C, per unit	0.65	(0.41, 1.02)	0.058	0.87	(0.51, 1.50)	0.622
Hypertension	1.26	(0.81, 1.98)	0.310			
Fatty liver disease	0.93	(0.51, 1.72)	0.822			
Combined subtype						
Age, per year	1.01	(0.99, 1.05)	0.349	1.01	(0.97, 1.05)	0.629
Disease duration, per year	0.93	(0.85, 1.02)	0.117	0.91	(0.83, 1.79)	0.316
BMI, per kg/m^2^	0.89	(0.81, 0.99)	0.024	0.86	(0.76, 0.98)	*0.018*
Serum urate, per mg/dL	0.84	(0.64, 1.11)	0.216	1.22	(0.83, 1.79)	0.316
Blood glucose, per mmol/L	0.92	(0.60, 1.43)	0.719			
eGFR, per ml/min/1.73 m^2^	1.00	(0.98, 1.02)	0.938			
TG/HDL-C, per unit	0.68	(0.42, 1.30)	0.142	0.42	(0.18, 0.96)	*0.039*
Hypertension	0.59	(0.30, 1.19)	0.141	0.60	(0.25, 1.43)	0.246
Fatty liver disease	1.82	(0.87, 3.79)	.0211			

*BMI* body mass index, *eGFR* estimated glomerular filtration rate, *TG/HDL-C* TG, triglyceride (mmol/L)/total high-density lipoprotein-cholesterol (mmol/L)

### Safety analysis

No serious adverse events were observed during the study. Gout flare rates were similar among the three groups, affecting 30 of 80 (37.5%) participants with renal overload type, 136 of 390 (34.9%) participants with underexcretion type, and 65 of 176 (37.4%) participants with combined type (Supplementary Table 2).

Liver function tests, kidney function, and markers of metabolic syndrome were monitored throughout the study (Supplementary Table 3). Increased ALT and AST were observed in participants with renal underexcretion type and combined type at 4, 8, and 12 weeks (*P* < 0.05). More participants with combined type required hepatoprotective treatment compared with overload type and underexcretion type (17.8% vs. 6.3% vs*.* 9.5% respectively, *P* = 0.005). There were no significant changes from baseline in serum creatinine or eGFR over the study period in any of the three groups. There were no participants withdrawn from the study because of eGFR < 30 ml/min/1.73 m^2^ (*P* = 0.413) and 2 participants (1 with underexcretion type and 1 with combined type) were withdrawn because of the aminotransferases elevated over three times the upper normal limit (Supplementary Table 2). Glu and TC decreased significantly in participants with renal overload type at week 4, 8, and 12 (*P* < 0.05).

## Discussion

This large prospective cohort study of gout patients commencing febuxostat indicates a potential role for assessing hyperuricemia clinical subtypes to guide prediction of febuxostat response. Overall, the proportion of participants achieving the SU target of < 6 mg/dL was similar in those with renal overload subtype and underexcretion subtype. However, those with combined subtype had lower serum urate responses, even after adjusting for potential confounders.

In this clinical gout cohort, the renal overload subtype was uncommon (12.4%), and participants with renal overload subtype had lower baseline SU level, which might explain the higher target-achieving rate and less febuxostat dose escalation in this subtype. One unanticipated finding was that although the baseline SU levels were comparable, participants with underexcretion subtype had better ULT response to febuxostat than those with combined subtype in terms of the SU target (< 6 mg/dL) achieving rate in the per-protocol analysis. In the intention-to-treat analysis, the SU target-achieving rates were similar between those with the two subtypes. There were 16.1% drop-out in the underexcretion subtype and 12.6% in the combined subtype, which may explain the difference. Furthermore, more participants with combined subtype required febuxostat dose escalation. Participants with combined subtype hyperuricemia had higher rates of metabolic syndrome (43.7%) and its components at baseline, including obesity, hypertension, hyperlipidemia, diabetes, and NAFLD. Additionally, in the participants with combined subtype hyperuricemia, BMI and high TG/HDL-C were independently associated with serum urate response. These data imply that insulin resistance is involved in mechanisms of combined subtype hyperuricemia and febuxostat response.

Numerous studies have shown associations between hyperuricemia and components of the metabolic syndrome [32]. Bidirectional Mendelian randomization analyses have confirmed that insulin resistance had an independent positive causal effect on SU concentrations [33]. Postulated mechanisms of this causal relationship include excessive de novo biosynthesis of purine nucleotide in dysfunctional adipose tissue and/or inflammation induced XO expression [19, 20]. Elevated insulin concentrations also enhance renal urate reabsorption via stimulation of urate-anion exchanger URAT1 [21] and the Na^+^-dependent anion co-transporter in brush border membranes of the renal proximal tubule [22]. Additionally, in the setting of insulin resistance, impaired oxidative phosphorylation may increase acidic metabolites, which are antagonists of renal urate excretion, and systemic adenosine concentration, which is the precursor of endogenous urate [22].

In this study, combined subtype hyperuricemia was associated with significantly lower serum urate responses to febuxostat, even after adjusting for BMI and baseline serum urate. These findings suggest that the attenuated serum urate responses to febuxostat with combined subtype hyperuricemia are not solely due to lower mg/kg doses of febuxostat or the need to reduce from a higher baseline serum urate level to achieve the serum urate target < 6 mg/dL. Febuxostat is primary cleared through entero-hepatic circulation, and it is possible that the high rates of NAFLD could have altered febuxostat metabolism. However, in univariable analysis, the presence of NAFLD did not associate with febuxostat response in the entire group or in those with combined subtype hyperuricemia.

Febuxostat can inhibit activity of ATP-binding cassette transporter G2 (ABCG2), the urate transporter mainly located in the intestinal tract and renal tubule, in a clinical dose by in vitro and in vivo studies [34]. ABCG2 dysfunction is a common mechanism of hyperuricemia resulting from decreased gut urate excretion [2–4]. Ichida et al. reported that the non-functional ABCG2 variants Q141K (rs2231142) and Q126X (rs72552713) were found in 45.9% and 4.1% of a Japanese group of hyperuricemia, respectively, and were associated with renal overload and combined type [35]. Similarly, the two non-functional ABCG2 variants in a Han Chinese group of gout were 49.6% and 4.7%, respectively [36]. Nonetheless, this might not be the case under the urate-lowering response of febuxostat in this study. ABCG2 is also recognized as one of the most important drug efflux transporters in the small intestine and may influence the bioavailability of many drugs. Unlike allopurinol, febuxostat is excreted both in the urine and in the feces. Whether febuxostat is also an ABCG2-substrate drug and whether ABCG2 inactivation increases the bioavailability of febuxostat itself is unknown. In fact, clinical studies have indicated that the rs2231142 Q141K was associated with poor response to allopurinol, but not to febuxostat [37, 38].

Several limitations of the study should be acknowledged. Firstly, since participants with febuxostat were only followed for 12 weeks, it was not possible to evaluate long-term efficacy or safety. Secondly, participants were all men recruited from a single medical center, and these results may not be generalizable to women or other countries. Thirdly, some patients with comorbidities were excluded to avoid confounding factors to the primary outcome in this study, which may limit the application in those patients. Expanding the results to patients with CKD ≥ stage 3 will be important in future studies. Finally, febuxostat was not titrated to the maximum approved dose. A long-term study in a larger population of multi-centered general gout patients with standard febuxostat titration procedure is needed to confirm the findings in this study.

In conclusion, this prospective cohort study identifies a role for pathogenic typing of hyperuricemia to predict treatment response to febuxostat in gout patients. We identify combined type hyperuricemia, which is strongly associated with features of metabolic syndrome, as a pathogenic type associated with lower rates of achieving serum urate target.

### Supplementary Information

**Additional file 1: Supplementary Table 1.** The low-purine diet. **Supplementary Table 2.** Adverse events during the study. **Supplementary Table 3.** Baseline clinical variables associated with the SU target achievement.

## Data Availability

The datasets used and analyzed during the current study are available from the corresponding author on reasonable request.

## Abbreviations

BMI: Body mass index
SBP: Systolic blood pressure
DBP: Diastolic blood pressure
TG: Triglyceride
HDL-C: High-density lipoprotein-cholesterol
ALT: Alanine aminotransferase
AST: Aspartate aminotransferase
eGFR: Estimated glomerular filtration rate
FEUA: Fractional excretion of urinary uric acid
UUE: 24-H urinary urate excretion
NAFLD: Non-alcoholic fatty liver disease
