# Supplementary material for: Response to febuxostat according to clinical subtypes of hyperuricemia: a prospective cohort study in primary gout
Source: Arthritis Res Ther. 2023 Dec 11;25:241. doi: 10.1186/s13075-023-03228-y (PMC10712161; doi:10.1186/s13075-023-03228-y)
Supplement: Supplementary file 1 — Additional file 1: Supplementary Table 1. The low-purine diet. Supplementary Table 2. Adverse events during the study. Supplementary Table 3. Baseline clinical variables associated with the SU target achievement. [file 13075_2023_3228_MOESM1_ESM.docx]

**Supplementary Table 1.** **The low-purine diet**

| Breakfast | Lunch | Dinner | Fruits |
| --- | --- | --- | --- |
| Flour 75 g | Rice 125 g or flour 125 g | Flour 75 g | 150 g |
| Vegetables 50 g | Two kinds of vegetables, 300 g in total | Vegetables 200 g |  |
| One egg or low-fat milk 250 mL | Pork, beef or chicken 30 g | Two eggs, or one egg and low-fat milk 250 mL |  |
|  | Blend oil 15 g | Blend oil 10 g |  |

**Supplementary Table 2 Adverse events during the study**

|  | **Renal overload** | **Underexcretion** | **Combined** |
| --- | --- | --- | --- |
| **Any gout flare, n (%)** | 30（37.50） | 136 (34.87) | 65 (37.35) |
| **One gout flare, n (%)** | 15（18.75） | 78 (20) | 41 (23.46) |
| **Two gout flares, n (%)** | 9（11.25） | 35 (8.97) | 12 (6.90) |
| **More than two gout flares, n (%)** | 6（7.5） | 23 (5.90) | 12 (6.90) |
| **Hepatoprotective treatment, n (%)** | 5 (6.25) | 37 (9.49) | 31 (17.81) *^#^ |
| **Aminotransferases >3*upper normal limit, n (%)** | 0 (0) | 1 (0) | 1 (0) |
| **Gastrointestinal adverse event, n (%)** | 0 (0) | 2 (0.01) | 1 (0.57) |
| **eGFR <30 ml/min/1.73m^2^,** **n (%)** | 0 (0) | 0 (0) | 0 (0) |

*, compared with overload type, *P*<0.05, ^#^, compared with underexcretion type, *P*<0.05.

**Supplementary Table 3 Biochemical parameters during the study**

|  | **Baseline** | **4 weeks** | **8 weeks** | **12 weeks** |
| --- | --- | --- | --- | --- |
| **Numbers of participants completed the follow up, N (%)** | | | | |
| Renal overload | 80 (100) | 73 (91.25) | 73 (91.25) | 71 (88.75) |
| Renal underexcretion | 390 (100) | 366 (93.85) | 334 (85.64) | 327 (83.85) |
| Combined | 174 (100) | 162 (93.10) | 154 (87.50) | 152 (87.93) |
| **Alanine aminotransferase, median (IQR), U/L** | | | | |
| Overload | 23 (19,30) | 26 (18,36) ^#^ | 23 (18,36) | 24 (18,35) |
| Underexcretion | 24 (17,34) | 27 (19,42) ^##^ | 27 (18,42) ^##^ | 26 (18,40) ^##^ |
| Combined | 29 (21,43) ** | 31 (22,52) *^##^ | 31 (23,48) **^##^ | 31 (22,47) **^##^ |
| **Aspartate aminotransferase median (IQR), U/L** | | | | |
| Overload | 21 (16,24) | 23 (18,28) | 22 (18,25) | 21 (18,24) |
| Underexcretion | 20 (17,25) | 22 (18,28) ^#^ | 23 (18,28) ^#^ | 22 (18,28) ^#^ |
| Combined | 21 (17,26) | 23 (19,29) ^#^ | 23 (18,27) ^#^ | 23 (19,28) *^#^ |
| **Blood glucose, mean (SD), mmol/L** | | | | |
| Overload | 6.02 (0.86) | 5.85 (0.67) ^#^ | 5.83 (0.73) ^#^ | 5.84 (0.64) ^#^ |
| Underexcretion | 5.69 (0.75) * | 5.63 (0.78) * | 5.61 (0.72) * | 5.66 (0.96) * |
| Combined | 5.77 (0.74) * | 5.75 (0.66) * | 5.59 (0.68) * | 5.58 (0.89) * |
| **Total cholesterol, mean (SD), mmol/L** | | | | |
| Overload | 4.99 (0.97) | 4.71 (0.90) ^#^ | 4.77 (1.00) ^#^ | 4.75 (0.97) ^#^ |
| Underexcretion | 4.98 (0.96) | 4.82 (0.95) | 4.88 (0.91) | 4.86 (1.03) |
| Combined | 4.97 (0.84) | 4.93 (0.93) | 4.95 (0.81) | 4.84 (0.99) |
| **Triglyceride, mean (SD), mmol/L** | | | | |
| Overload | 1.78 (1.00) | 1.75 (0.93) | 1.82 (1.36) | 1.75 (0.92) |
| Underexcretion | 2.11 (1.49) | 2.02 (1.42) | 1.95 (1.13) | 1.90 (1.23) |
| Combined | 2.32 (1.51) * | 2.19 (1.40) * | 2.26 (1.33) * | 2.08 (1.25) * |
| **Creatinine, mean (SD), *μ*mol/L** | | | | |
| Overload | 86.46 (13.26) | 85.69 (15.82) | 85.14 (12.72) | 85.16 (13.02) |
| Underexcretion | 87.58 (12.64) | 85.69 (15.20) | 85.55 (15.67) | 85.60 (17.35) |
| Combined | 83.80 (12.65) | 84.11 (15.28) | 84.06 (13.62) | 84.27 (16.22) |
| **Estimated glomerular filtration rate, mean (SD)，ml/min/1.73 m^2^** | | | | |
| Overload | 89.92 (33.91) | 90.34 (16.91) | 90.82 (20.21) | 90.91 (15.40) |
| Underexcretion | 91.54 (15.52) | 93.44 (19.33) | 93.59 (19.85) | 92.14 (19.69) |
| Combined | 96.81 (17.69) * | 98.10 (27.41) * | 96.86 (18.77) * | 94.86 (17.91) |

*, compared with combined group, *P* <0.05; **, compared with combined group, *P* <0.001; ^#^, compared with baseline, *P* <0.05; ^##^, compared with baseline, *P* <0.001.
